# Has the Early Majority Been Reached? Specialised Inpatient Palliative Care Utilisation in Common Solid Tumours in Germany

**DOI:** 10.3390/cancers18193163

**Published:** 2026-10-01

**Authors:** Marcel A. Kamp, Martina Kern, Nazife Dinc, Birgitt van Oorschot, Lukas Radbruch, Christiane von Saß

**Affiliations:** 1 Faculty of Health Sciences Brandenburg, Brandenburg Medical School Theodor Fontane, 15562 Rüdersdorf bei Berlin, Germany; 2 Immanuel Clinic Rüdersdorf, Universitätsklinikum der MHB, 15562 Rüdersdorf bei Berlin, Germany; 3 ALPHA Rheinland, 53111 Bonn, Germany; 4 Department of Neurosurgery, Jena University Hospital, Friedrich-Schiller University, 07747 Jena, Germany; 5 Independent Researcher, 15834 Rangsdorf bei Berlin, Germany; 6 Department of Palliative Medicine, University Hospital Bonn, 53127 Bonn, Germany

**Keywords:** palliative care, early integration, cancer, solid cancer, diffusion of innovation, innovation, future healthcare

## Abstract

**Background:** Randomised trials indicate that early integration of specialised palliative care improves quality of life and, in some studies, survival in advanced cancer, yet its implementation in inpatient oncology remains inconsistent. This study quantifies the coded utilisation of specialised inpatient palliative care in German hospitals and interprets adoption patterns using the diffusion of innovations theory. **Methods:** We conducted a cross-sectional analysis of nationwide hospital cases reimbursed under the German diagnosis-related groups (DRG) system in 2023. The analysis included the most common solid malignancies and assessed the utilisation of specialised inpatient palliative care overall and stratified by metastatic status, tumour entity, comorbidities, and treating medical specialties. **Results:** Specialised inpatient palliative care was provided in 5.4% of hospital cases involving the most common malignant tumours. Among patients with organ metastases, utilisation increased to a median rate of 15.0% of hospital cases, with the highest rates observed in cases with brain (17.8%), bone (17.0%), and pleural metastases (16.1%). Palliative care involvement correlated positively with in-hospital mortality. Interpreted cautiously through the diffusion of innovations theory, which frames the 15 to 20% band as a heuristic landmark rather than a validated threshold, utilisation even in the metastatic subgroup reaches only the lower edge of this band, indicating that uptake remains confined to innovators and early adopters and has not extended to the early majority. **Conclusions:** Specialised inpatient palliative care remains underutilised in German oncology, reaching 5.4% of all cases and a median of 15.0% even in metastatic disease. Interpreted cautiously through the diffusion of innovations theory, which frames the 15 to 20% band as a heuristic landmark, uptake appears confined to early adopters and has not yet reached the early majority.

## Simple Summary

Patients with advanced cancer benefit when specialised palliative care starts early. It relieves symptoms, supports patients and families, and improves quality of life. We examined how often German hospitals actually provide this care to patients with common solid cancers. We analysed nationwide hospital billing data from 2023. Only about 5 in 100 hospital stays for these cancers included specialised palliative care. When the cancer had spread to other organs, this rose to about 15 in 100. Rates were highest for spread to the brain and the bones. We used a model that describes how new practices spread through a health system. By this model, palliative care is still used mainly by pioneers and has not yet reached routine practice. Many patients with advanced cancer in Germany may therefore miss care that could help them.

## 1. Introduction

Palliative medicine aims to improve the quality of life of patients and their families. It focuses on the prevention, identification, and relief of suffering across physical, psychological, social, and spiritual domains [1]. During its early years, palliative care focused on end-of-life support [2]. Over time, the concept of improving quality of life and alleviating suffering expanded beyond care limited to the terminal phase after cessation of life-prolonging treatment. Under the concept of early integration, palliative care now accompanies patients at earlier stages of disease in parallel with tumour-directed therapies. Studies by Jennifer Temel and colleagues demonstrated that early palliative care not only improves quality of life but may also positively influence survival [3,4,5]. Although subsequent studies did not consistently reproduce this survival benefit, the concept gained substantial momentum and became widely incorporated into clinical guidelines and recommendations [6,7]. The 2024 guideline update of the American Society of Clinical Oncology (ASCO) explicitly recommends an early integration of palliative care into the treatment of patients with advanced cancer [8], generally defined as an initiation of palliative care within 8–12 weeks after diagnosis [8,9,10].

This raises the question of how widely specialised inpatient palliative care utilisation for all patients with palliative oncological diagnoses, together with early palliative care integration, has diffused into routine clinical practice. Everett M. Rogers’ diffusion of innovations theory explains how new ideas and technologies spread within social systems and under what conditions they are adopted or rejected [11]. The theory identifies five core attributes that shape adoption, which are relative advantage, compatibility, complexity, trialability, and observability. Rogers further distinguishes adopter groups according to their timing of adoption, namely innovators (approximately 2.5% of potential users), early adopters (approximately 13.5%), the early majority (approximately 34%), the late majority (approximately 34%), and laggards (approximately 16%) [11]. These proportions derive from partitioning a normal distribution of adoption times into standard deviation segments and are therefore schematic rather than empirically fixed boundaries. Innovators and early adopters together constitute only about 16% of users, yet they are thought to act as opinion leaders who legitimise innovations within the wider system [11]. Diffusion typically accelerates once adoption extends beyond early adopters into the early majority, a transition Berwick, drawing on Rogers, described as often occurring at approximately 15–20% adoption [12]. This range is a widely cited heuristic, not a threshold empirically validated for health service innovations. Rogers derived it from general diffusion research in fields such as agriculture and consumer goods, and Berwick framed it explicitly as an approximation rather than a fixed parameter [11,12,13]. We therefore adopt it as a theory-derived landmark for exploratory interpretation only.

Against this background, the aim of the present study is to quantify the proportion of inpatient palliative care services reimbursed within the German diagnosis-related groups (DRG) system for the most common oncological diseases. We subsequently interpret these findings in light of the diffusion of innovations theory [14,15].

## 2. Methods

### 2.1. Ethical Approval and Data Accessibility

The institutional and local ethics committees at Friedrich Schiller University Jena (Study ID: 2025-3696-BO-D) and Brandenburg Medical School, Germany (Study ID: 190032024-ANF) approved the data analysis. The reporting follows the STROBE guidelines for observational studies [16].

### 2.2. Study Design, Setting, and Data Sources

We conducted a cross-sectional analysis of routinely collected nationwide administrative hospital data from all hospital cases in Germany in 2023. The dataset was derived from routine administrative hospital data collected under §21 of the German Hospital Remuneration Act and was provided by the Institute for the Remuneration System in the Hospital Sector (InEK GmbH, Siegburg, Germany). The data underlying this study were obtained from the InEK Datenbrowser (https://datenbrowser.inek.org/), last accessed on 12 November 2025. The extracted case-level tables and analysis files are available from the corresponding author upon reasonable request.

### 2.3. Cohort Definition, Participants, and Study Size

The study aimed to analyse the frequency of palliative care involvement within the German DRG system among patients with common solid malignancies [17]. We defined common solid tumours as those accounting for more than 3% of all newly diagnosed cancer cases in Germany in 2023 [17]. These tumour entities included malignancies of the breast, prostate, pancreas, lung, colorectum, uterine corpus, cervix, stomach, ovary, kidney, bladder, oral cavity and pharynx, as well as malignant melanoma. We restricted the analysis to common solid malignancies because haematological malignancies follow fundamentally different, often less predictable trajectories, with a considerably higher proportion being curable or running a chronic course over years to decades [18,19]. They lack an analogue to the organ metastasis and TNM framework underpinning our central subgroup analysis and are frequently disseminated, e.g., in the bone marrow at diagnosis [20,21]. On the same grounds, a landmark early-palliative-care trial explicitly excluded them because of their less predictable course [22]. Although current guidance now recommends early palliative care for haematological malignancies as well, the supporting randomised evidence remains limited to acute myeloid leukaemia and stem-cell transplantation, whereas the diffusion concept we assess is anchored in the mature solid-tumour evidence base [8,23,24]. Combining these heterogeneous entities would confound the case-based diffusion interpretation and is better addressed in disease-specific analyses [23].

Based on this definition, we applied the following inclusion criteria: (1) A diagnosis of one of the specified solid tumours as either a primary or secondary diagnosis, (2) hospital treatment during 2023 and (3) age 18 years or older. Owing to technical constraints of the dataset, the analysis focused on hospital cases rather than individual patients, and individual-level longitudinal data were unavailable. The study population therefore comprised all hospital cases meeting these criteria in 2023. The dataset permits retrieval of case counts and treatment counts stratified by tumour entity, but does not allow linkage of multiple diagnoses to a single case. Consequently, cases with several primary cancers or metastatic sites cannot be individually identified or excluded, and each case is attributed to the tumour entity or entities recorded.

To calculate the number of cases for a particular diagnosis, we retrieved the counts of hospital cases of each cohort (e.g., defined by the tumour type, combinations of diagnoses or the combination of tumour type and medical speciality), where the diagnosis was listed as either primary, secondary diagnoses or as both primary and secondary diagnoses. To avoid counting a case twice when a diagnosis appeared as both a primary and secondary diagnosis, we subtracted the number of double-coded cases from the sum of cases in which the ICD-10-GM code appeared as either the primary or secondary diagnosis. This calculation addresses the double-coding within a single case, distinct from duplicate reporting across cases. Duplicate reporting of hospital cases is precluded by the InEK reimbursement structure, as each case is billed and reported only once. However, multiple hospital admissions of the same patient within the same calendar year are retained as separate cases, consistent with the case-based nature of DRG data.

### 2.4. Definitions and Variables

All diagnoses, in particular primary tumours and organ metastases, were identified and defined using the International Statistical Classification of Diseases and Related Health Problems, 10th Revision, German Modification (ICD-10-GM, Supplementary Table S1) [25]. In the German DRG system, each case is assigned a single primary diagnosis and may have multiple secondary diagnoses. Medical procedures and treatments were identified using their corresponding procedural codes (*Operationen- und Prozedurenschlüssel, OPS*) [26]. The selection of analysed comorbidities was guided by the Charlson Comorbidity Index [27]. The individual specialist disciplines (e.g., oncology, radio-oncology, …) were identified based on their respective codes from Annex 1 of the Federal Nursing Ordinance within the Federal Nursing Fee Ordinance of 31 December 2003. Specialty was assigned using the InEK Datenbrowser’s Fachabteilung selection filter, which identifies all cases with a valid contact to the selected specialty at any point during the hospital stay. Cases with an internal transfer between departments are consequently associated with more than one specialty and appear in the case counts for each specialty involved, so that specialty stratified case counts are not mutually exclusive across specialties.

Specialised palliative care was identified using three OPS codes. OPS code 8-982 (complex palliative care treatment) is delivered under specialist management with additional qualification in palliative medicine, standardised basic assessment, weekly interdisciplinary team meetings, and multi professional involvement. OPS code 8-98e (specialised inpatient complex palliative care treatment) requires an independent palliative care unit, a 24/7 qualified on-call service, and medical and nursing leadership each with at least six months of specialist palliative care experience. OPS code 8-98h (specialised palliative care inpatient consultation service) applies comparable structural criteria to 8-98e but is delivered as a consultation liaison service to other wards.

The mortality rate was defined as the proportion of fatal hospital cases relative to the total number of cases within the corresponding cohort. Fatal hospital cases are identified by querying the coded discharge reason documented according to Section 301, Paragraph 1, Sentence 1 of the German Social Code Book V (discharge reason 07: death).

### 2.5. Addressing Bias

We minimised selection bias by including all German hospital cases with the analysed tumours treated in 2023 that were billed within the DRG system, yielding a comprehensive national sample. In accordance with InEK data protection regulations, diagnosis and procedure codes representing fewer than four cases are not displayed in the Datenbrowser and cannot be retrieved, disaggregated, or reconstructed from adjacent categories. This suppression applies at the level of individual diagnosis or procedure codes rather than to the dataset as a whole, and affects only small subgroups. It does not permit imputation or estimation of the true underlying values. The lack of referral timing data introduces a potential misclassification bias, as reported utilisation may include both early and terminal-phase palliative care under the same code. The analysis was limited to hospital cases rather than individual patients. The billing codes for specialised palliative care treatment from palliative care units that bill outside the DRG system cannot be captured. This particularly concerns the billing code for specialised palliative care treatment in a palliative care unit (8-98e) of approximately 70 palliative care units [28,29]. A sensitivity analysis estimating the potential ceiling effect of this gap is reported in the Limitations. Measurement bias was reduced by identifying palliative and intensive care treatments using predefined OPS and ICD-10 codes, ensuring standardised case identification. Although some misclassification is possible, errors are expected to be minimal in billing data. Key clinical information, including treatment timing, integration of specialised inpatient palliative care, and patient-reported outcomes, was unavailable. To account for disease severity, relevant comorbidities were included, and a separate analysis of patients who died in hospital was performed.

### 2.6. Data Management and Statistical Analysis

Data were extracted from the InEK data browser and organised using Microsoft Excel for Mac (Version 16.93, Microsoft Corporation, Redmond, WA, USA). GraphPad Prism 9 for macOS (Version 9.5.1, GraphPad Software, La Jolla, CA, USA) was used for statistical analysis and visualisation. Two units of analysis were used and are reported separately. First, we calculated unadjusted odds ratios from aggregated 2 × 2 contingency tables of case counts [16,30,31]. For each group, the group’s case counts with and without specialised palliative care were compared against the corresponding counts for all remaining cases, so that each tumour entity, metastatic site, or specialty was compared against the remainder of the cohort rather than against the entire cohort, since the latter would include the group under comparison and bias estimates toward the null. The standard error of ln(OR) was obtained from the four cell counts. Because the data were available only as aggregated case counts, no individual-level logistic regression was performed. The odds ratios are therefore unadjusted and do not account for age, sex, or comorbidity. Second, linear regressions relating in-hospital mortality to specialised palliative care frequency were performed at the aggregate level, with each data point representing one tumour entity, one metastatic site, or one medical specialty, not individual hospital cases. These associations are therefore ecological and should not be interpreted at the patient level. Regressions were unweighted, so each entity contributed equally regardless of case volume. We chose a significance level α of 5% (0.05) for the present study and performed in total three statistical evaluations (κ = 3). To adjust for multiple comparisons, Šidák’s correction was applied:

$$\alpha_{adjusted}=1-{\left(1-\alpha \right)}^{1/\kappa }$$

A significance level of <0.017 was used [32].

## 3. Results

In 2023, German hospitals recorded 15,200,893 adult patient hospitalisations. Solid malignant tumours featured in 1,637,202 (11%) of these hospital cases. A malignant solid tumour was the primary diagnosis in 1,243,449 cases, the secondary diagnosis in 687,690, and both the primary and secondary diagnoses in 293,937 cases.

The analysis focused on solid malignant tumours with an incidence of ≥3% in Germany, accounting for 1,292,726 cases, or 77% of all cases involving patients with a solid malignant tumour. Females comprised 47.1% (608,178 cases) and males 52.9% (684,474 cases) of these cases (74 cases involved unknown or diverse gender). Cases with patients aged ≥65 years represented 64.9% (839,533 cases), while those with patients aged 18 to 29 years accounted for 0.31% (4043 cases). The most common solid tumours were malignant lung tumours (254,444 cases; 19.7% of the analysed tumour cases), breast tumours (207,681 cases; 16.1%), and colorectal tumours (181,464 cases; 14%). Malignant cervical tumours generated the fewest cases in this group (18,215 cases). Among the 1,292,726 cases examined, clinicians assigned 884,224 ICD-10 codes across 877,750 cases for lymph node or organ metastases and 631,972 codes for organ metastases alone, allowing for multiple codes per case. The most frequent organ metastases were located in the liver and intrahepatic bile duct (11.3% of examined cases), bone or bone marrow (10.2%), lung (7.5%), peritoneum and retroperitoneum (5.2%), and cerebral metastases/leptomeningeal carcinosis (4.9%). As anticipated, different tumour entities exhibited distinct metastasis patterns.

**Figure 1:**
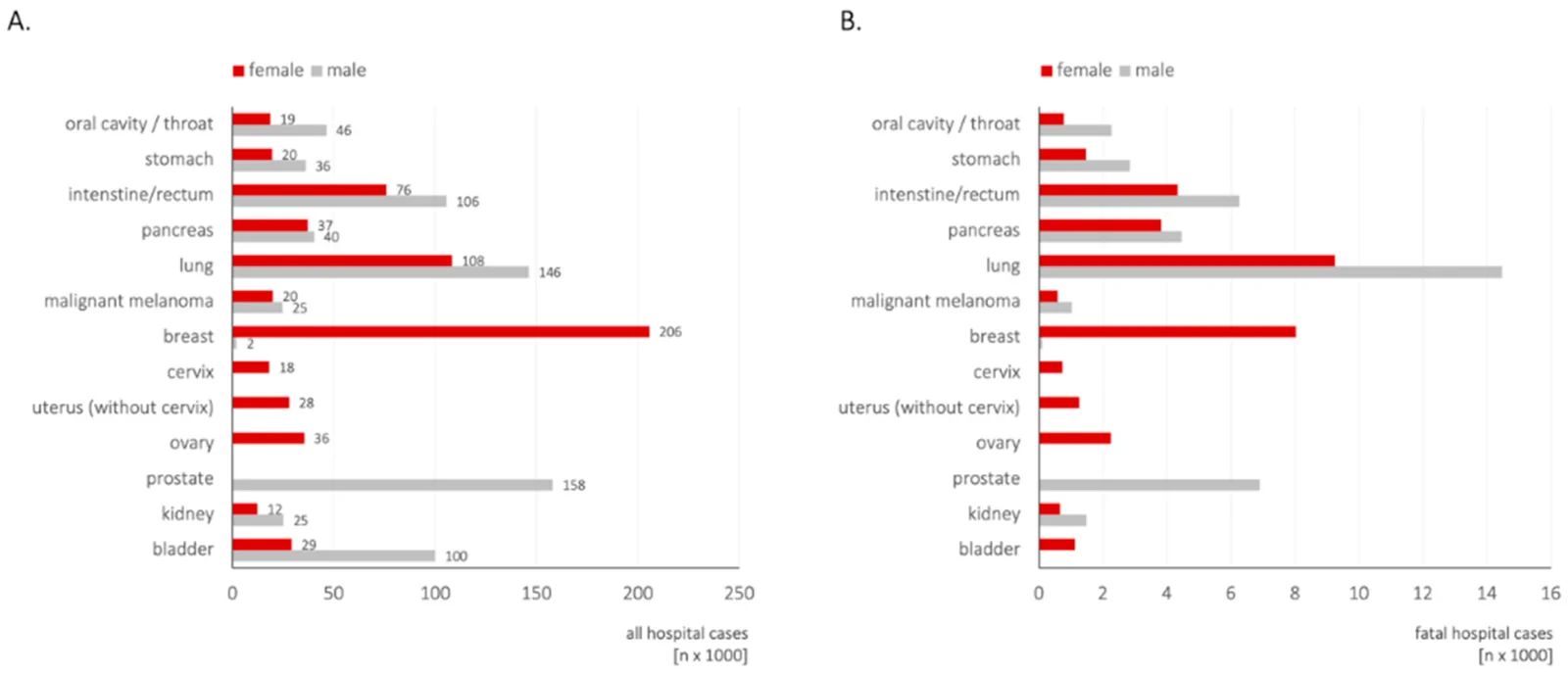
Gender distribution. Gender distribution across the analysed tumour entities in the total study cohort (**A**) and among hospital cases resulting in in-hospital death (**B**).

Table 1 and the accompanying Figure 1 provide detailed age and sex distribution information for each tumour type.

### 3.1. Specialised Palliative Care Involvement

**Figure 2:**
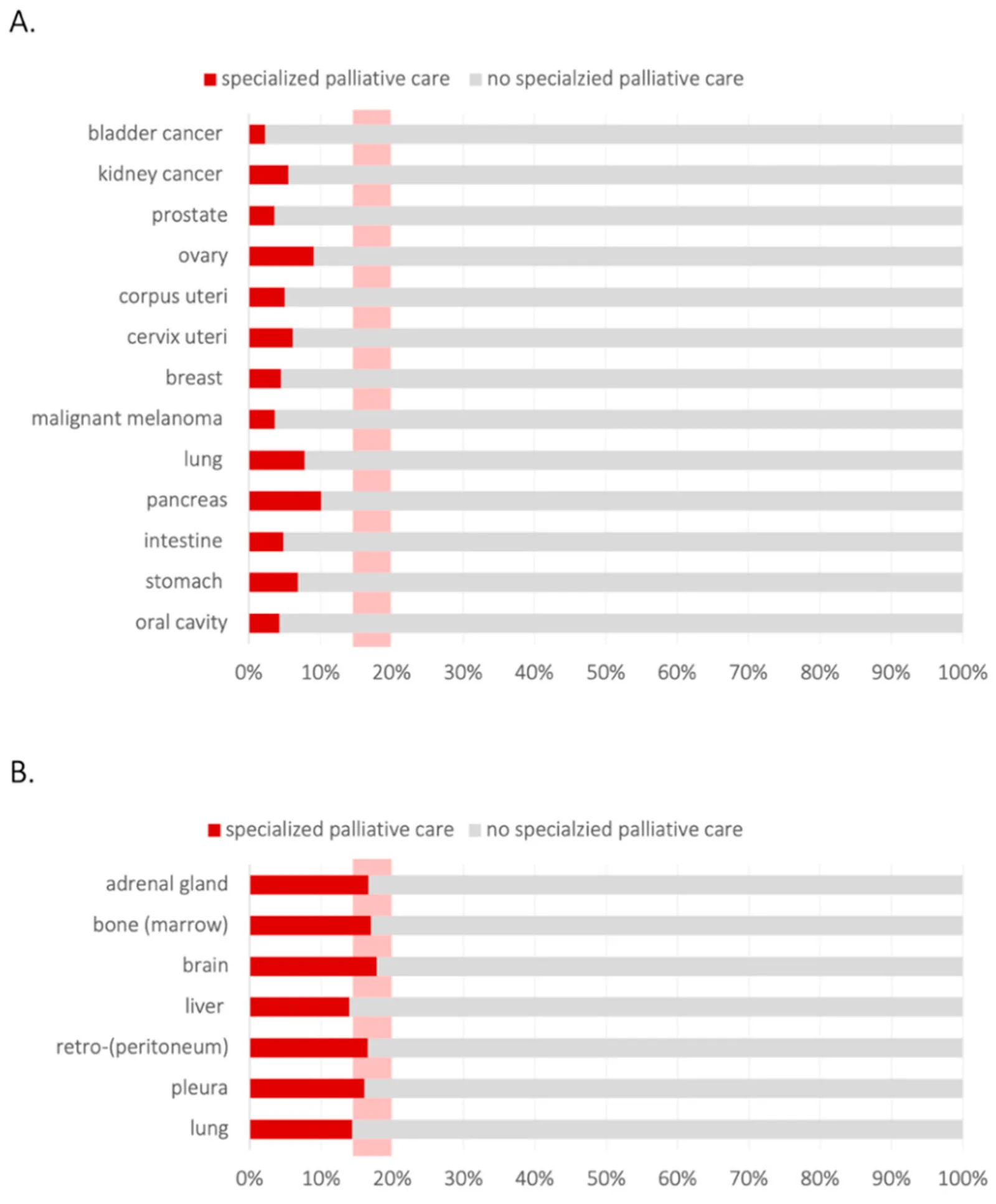
Frequency of palliative care involvement in hospital cases with oncologic patients. Panel (**A**) shows the proportion of hospital cases that included specialised palliative care involvement care as defined by OPS codes 8-982, 8-98e, and 8-98h. Panel (**B**) depicts the proportion of hospital cases of patients with frequent organ metastases that included specialised palliative care involvement. The shaded pink area indicates the tipping-point range described by diffusion of innovations theory (approximately 15–20% adoption), beyond which acceptance extends from early adopters to the early majority and diffusion becomes self-sustaining.

Specialised palliative care was delivered in 70,382 of the 1,292,726 hospital cases analysed, corresponding to an overall rate of 5.4%. In these cases, hospitals recorded a total of 71,698 specialised palliative care procedure codes, including 21,917 codes (31.1% of 70,382 cases) for complex palliative care, 34,219 codes (48.6%) for specialised complex treatment on a palliative care unit, and 15,562 codes (21.7%) for palliative care consultation services (Table 2, Figure 2, Supplementary Figure S1).

**Figure 3:**
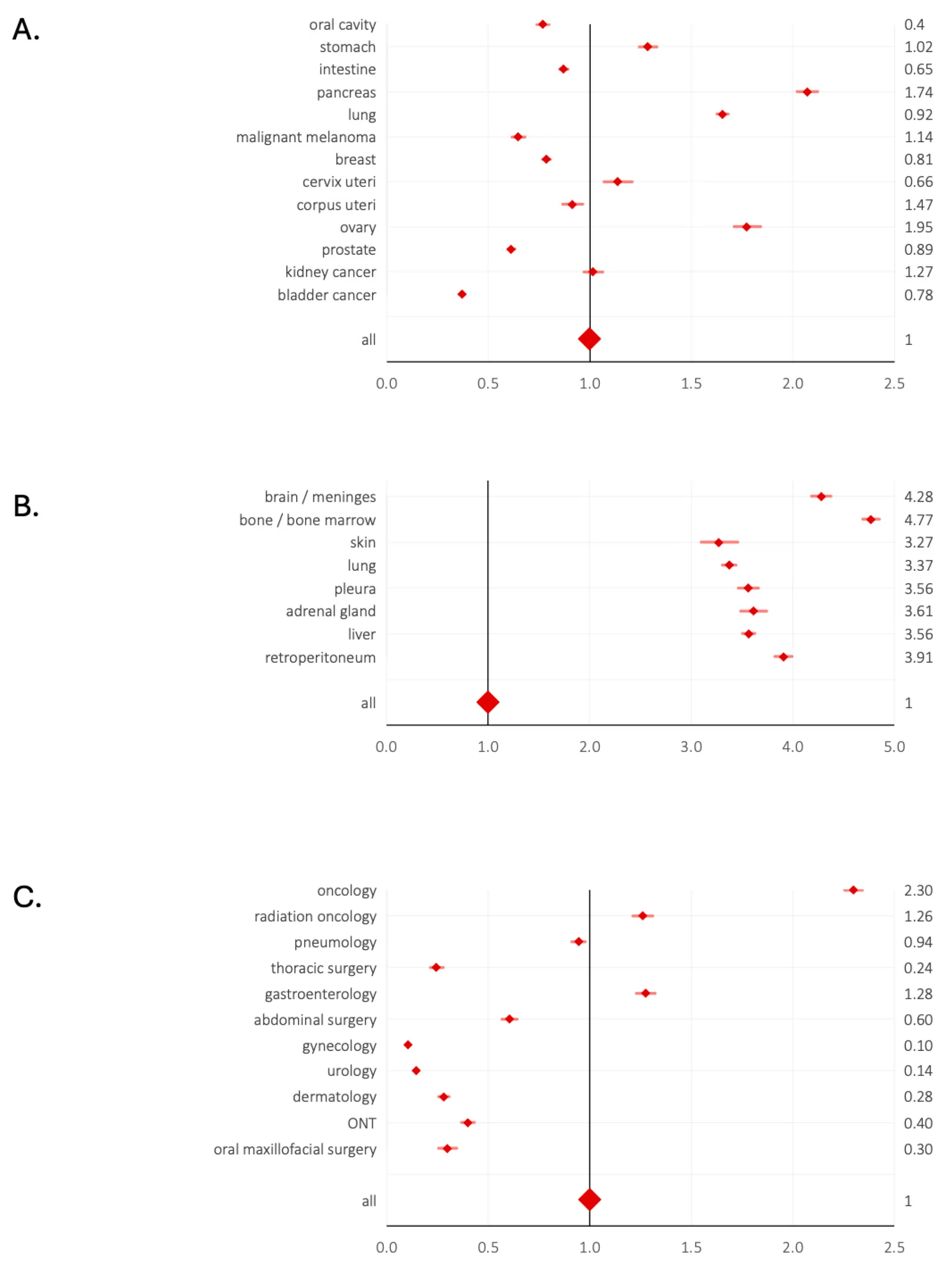
Odds ratios for specialised palliative care utilisation. This figure presents odds ratios for specialised palliative care treatment according to tumour entities (**A**), presence of metastases (**B**), and treating medical specialties (**C**). Odds ratios are calculated relative to the overall frequency of specialised palliative care utilisation in the entire analysed cohort (OR = 1). Abbreviations: OR, odds ratio.

Patients with pancreatic malignancies showed the highest utilisation of specialised palliative care (10.1% of all cases), whereas patients with bladder cancer showed the lowest rate (2.2%). Across the analysed tumour entities, the median utilisation rate reached 5% (interquartile range [IQR]: 4.3–6.8%). We calculated odds ratios (OR, Figure 3, Supplementary Table S4) for the use of specialised palliative care relative to all analysed hospital cases. Patients with pancreatic cancer (OR: 2.07; 95% CI: 2.02–2.12), ovarian cancer (OR: 1.77; 95% CI: 1.71–1.84), and lung cancer (OR: 1.65; 95% CI: 1.63–1.68) showed the highest odds of specialised palliative care involvement, calculated relative to the remainder of the cohort. Detailed rates and odds ratios of specialised palliative care involvement for each primary tumour entity in the presence of organ metastases are provided in Supplementary Table S3.

As the analysed tumour cases also included patients treated with curative intent, we additionally analysed the subgroup of hospital cases involving organ metastases, in which specialised palliative care needs are generally more pronounced. Across all analysed primary tumours and metastatic sites, the median rate of specialised palliative care involvement reached 15.0% (interquartile range [IQR]: 8.3–19.4%). Among hospitalised cases involving one of the analysed tumours with additional cerebral metastases, specialised palliative care involvement reached 17.8%. Corresponding rates amounted to 17.0% for bone metastases, 16.1% for pleural metastases, and 14.3% for lung metastases. The presence of cerebral metastases markedly increased the likelihood of specialised palliative care involvement (OR: 4.3; 95% CI: 4.2–4.4). Bone metastases (OR: 4.8; 95% CI: 4.7–4.9), pleural metastases (OR: 3.6; 95% CI: 3.5–3.7), (retro-) peritoneal metastases (OR: 3.9; 95% CI: 3.8–4), and lung metastases (OR: 3.4; 95% CI: 3.3–3.4) showed similarly elevated odds.

Next, we analysed the frequency of specialised palliative care involvement across medical specialties that commonly serve as the primary treating disciplines in oncological centres. Haematology/oncology demonstrated the highest rate of specialised palliative care involvement (10.4%), followed by gastroenterology (6.8%) and radiation oncology (6.7%). In contrast, gynaecology (0.7%) and urology (1.0%) showed the lowest rates. Accordingly, the odds ratios, calculated relative to the remainder of the cohort, ranged from 2.30 (95% CI: 2.25–2.34) for haematology/oncology to 0.10 (95% CI: 0.10–0.11) for gynaecology and 0.14 (95% CI: 0.14–0.15) for urology. These 11 specialties, selected as the primary treating disciplines in oncological centres, accounted for 30,701 of the 70,382 total specialised palliative care cases (43.6%).

### 3.2. Comorbidities

The modified Charlson Comorbidity Index assessed comorbidities, which frequently co-occurred in these oncological hospital cases. Excluding the codes for the primary solid malignancies (C00–C76), the 1,292,726 hospital cases showed 1,691,508 codes for a Charlson Comorbidity Index-associated comorbidity (Supplementary Table S2). Uncomplicated diabetes occurred in 15.4% of cases, lung disease in 11.2%, kidney disease in 10.4%, and acute myocardial infarction in 2.8%. As expected, pancreatic malignancies demonstrated high diabetes frequencies (31%), lung malignancies showed high rates of lung disease (30%), and cervical cancer higher rates of HIV infections (0.2%). Among comorbidities captured by the Charlson Comorbidity Index, severe liver disease showed the strongest association with specialised palliative care involvement (OR: 5.4; 95% CI: 4.98–5.84), followed by paraplegia (OR: 3.1; 95% CI: 2.89–3.21), heart failure (OR: 1.64; 95% CI: 1.6–1.68), and dementia (OR: 1.46; 95% CI: 1.39–1.53, Supplementary Figure S2).

### 3.3. In-Hospital Mortality Rate

A total of 77,025 of the 1,292,726 analysed hospital cases ended in death during the hospital stay, yielding an overall in-hospital mortality rate of 6% (Supplementary Table S3). The median in-hospital mortality rate across the examined tumour entities was 4.7%. Malignant pancreatic tumours had the highest rate at 10.7%, and bladder malignancies had the lowest at 3.2%. Mortality increased significantly when clinicians diagnosed organ metastases. The median mortality rate for lung metastases was 12.5% (range: 4.1–15.5%), 18.4% for pleural metastases (range: 6.2–24.2%), 16.6% for liver metastases (range: 3.9–19.8%), and 14.3% for cerebral and/or leptomeningeal metastases (range: 10.1–20.3%).

**Figure 4:**
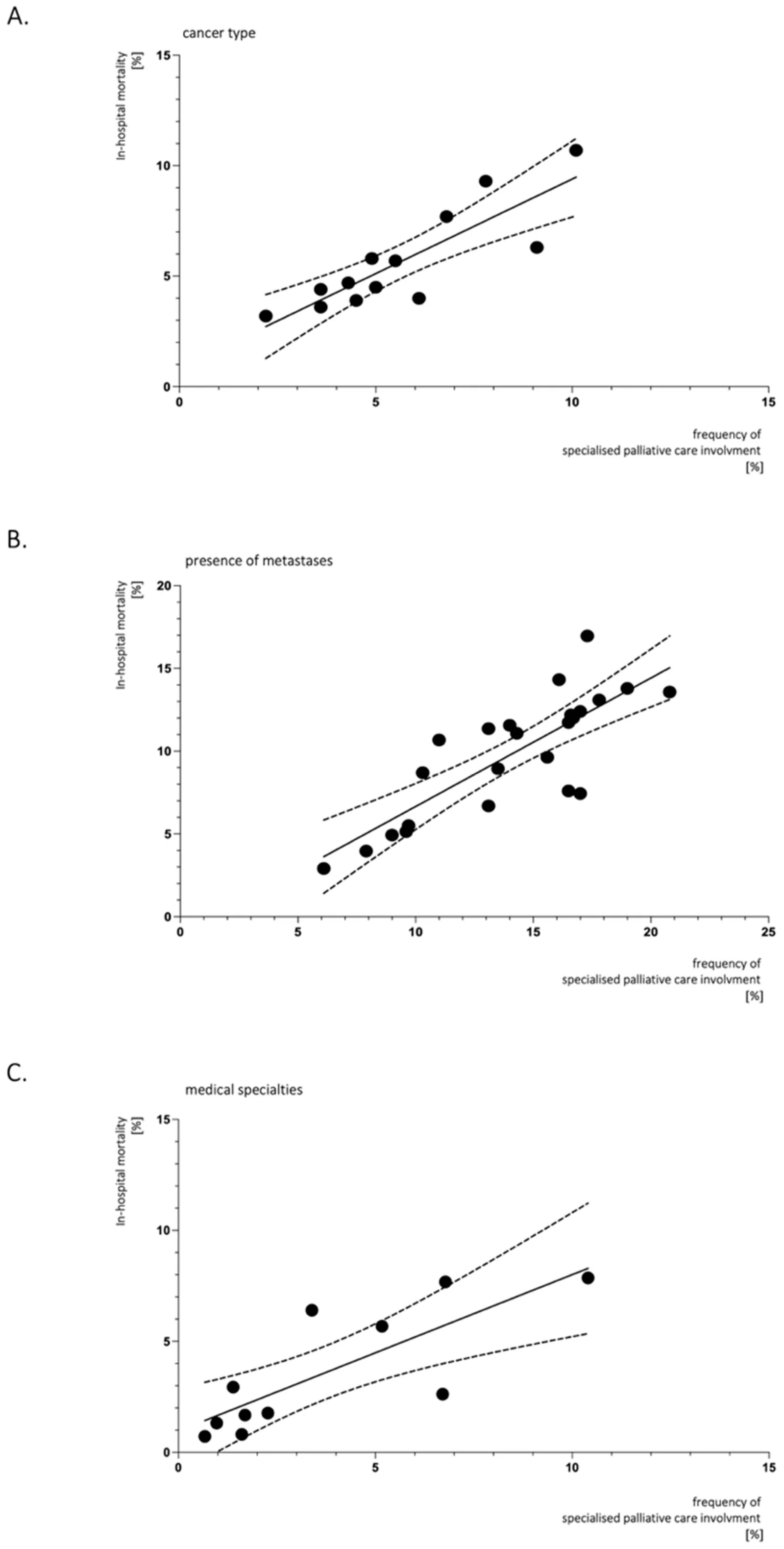
Linear regression analyses. Linear regression analyses for the association between in-hospital mortality and the frequency of specialised palliative care involvement for different tumour types (A.: y = 0.86x + 0.84; R^2^ = 0.72; F = 28.5; *p* = 0.0002), in cases with the presence of metastases (B: y = 0.78x − 1.1; R^2^ = 0.65; F = 41.4; *p* < 0.0001) and across different medical specialties (C: y = 0.70x + 0.96; R^2^ = 0.64; F = 15.7; *p* = 0.003). Each data point represents one tumour entity (**A**), metastatic site (**B**), or medical specialty (**C**). Associations are ecological and do not permit patient-level inference. The dashed lines represent the 95%-CT.

Linear regression analysis revealed a significant association between in-hospital mortality and the frequency of specialised palliative care involvement across the analysed tumour entities (y = 0.86x + 0.84; R^2^ = 0.72; F = 28.5; *p* = 0.0002, Figure 4), across metastatic tumour entities (y = 0.78x − 1.1; R^2^ = 0.65; F = 41.4; *p* < 0.0001), and across medical specialties (y = 0.70x + 0.96; R^2^ = 0.64; F = 15.7; *p* = 0.003). As these regressions are based on entity-level aggregates rather than individual patients, they reflect ecological associations and should not be interpreted as evidence of a relationship between specialised palliative care and mortality at the patient level.

Of the 77,025 hospital cases that ended in death, 24,566 (31.9%) involved patients receiving specialised palliative care (Table 2).

## 4. Discussion

Our analysis quantified the proportion of inpatient palliative care services reimbursed under the German diagnosis-related groups (DRG) system for the most common oncological diseases and yielded the following key findings.

1. Specialised palliative care was provided in 5.4% of hospital cases involving the most common malignant tumours in Germany.
2. The rate of inpatient palliative care treatment showed a positive ecological correlation with hospital mortality across tumour entities and specialties.
3. Among hospital cases with one of the analysed tumours and organ metastases, the median rate of specialised palliative care involvement reached 15.0%. This rate should be regarded as an upper bound on true early palliative care integration, not its direct measure. Projected onto Rogers’ adopter category distribution, and treating the 15 to 20% band as a heuristic landmark rather than a validated threshold, this level corresponds to acceptance by innovators and early adopters only. Adoption by the early majority has not yet occurred.

In Germany, the rate of specialised inpatient care for the malignancies examined with an incidence ≥ 3% is 5.4%. This level remains well below the heuristic 15 to 20% band described in the diffusion of innovations theory. These tumour cases include patients receiving curative treatment. Patients with organ metastases are highly likely to require palliative care, and clinical guidelines and recommendations explicitly advocate early palliative care integration and co-treatment for these groups [6,7,8,9]. However, metastatic status alone does not define treatment intent. Selected patients with oligometastatic disease may still benefit from intensive multimodal approaches with curative or disease-controlling aim [33,34]. The Lancet Oncology Commission (2025) identifies a profound “human crisis” in global cancer care, noting that current systems frequently neglect the emotional, relational, and existential dimensions of the patient experience despite significant biomedical progress [35]. This crisis stems from structural failures, including an over-reliance on technology, fragmented delivery models, and economic incentives that prioritise clinical throughput over compassionate, person-centred communication. To resolve this dehumanisation, the Commission advocates for the immediate, universal integration of palliative and psychosocial care, positioning empathetic engagement not as an elective supplement but as a fundamental requirement of oncological practice [35]. Across all analysed primary tumour entities and metastatic sites in the present study, the median utilisation of specialised palliative care reached 15.0%. The highest rates of inpatient specialised palliative treatment occurred in hospital cases with brain metastases (17.8%), bone metastases (17.0%), and pleural metastases (16.1%). Because the InEK dataset does not capture the timing of palliative care referral relative to diagnosis, the reported utilisation rate cannot distinguish early integration from palliative care initiated shortly before death. Given that 31.9% of hospital deaths in this cohort involved specialised palliative care coding, a substantial but unquantifiable proportion of the reported utilisation likely reflects late-stage or end-of-life care rather than early integration. The observed utilisation rate should therefore be regarded as an upper bound on the true rate of early palliative care integration, not as its direct measure.

Our findings align closely with our previous work on glioblastomas and cerebral metastases [28,29]. A German study based on statutory health insurance data from the state of Baden-Württemberg identified 3535 patients with advanced cancer who required palliative care in 2015. Of these patients, 669 (18.9%) were referred to specialised outpatient palliative care teams, and 45.1% of those referrals involved early integration of palliative care [36]. Earlier published data highlighted the gap between patient needs and actual service delivery. The analysis of health insurance records for 80,768 individuals revealed that only 7.8% of cancer patients accessed specialised outpatient palliative care in their final year, while the system provided just 8.4 complex palliative treatments per 10,000 inhabitants [37]. Another study analysing health insurance data on specialised palliative care indicate that approximately one third of all patients receive specialised palliative care during the last year of life [38,39]. However, comparative European analyses classify Germany, the Netherlands, the United Kingdom, and Switzerland as countries with a relatively high level of palliative care integration [40]. International data nevertheless reveal substantial variation in the timing of palliative care delivery. In Victoria, Australia, 66% of patients who died from cancer in 2018 received palliative care, and 18% accessed palliative care at least three months before death [41]. The median interval between first palliative care contact and death was 22 days [41]. In Canada, 12.6% of inpatients received some form of palliative care during the year preceding death [42]. In the United States, 8.0% of patients with newly diagnosed metastatic cancer received early specialised palliative care within eight weeks of diagnosis in 2018 and 2019 [43]. Palliative care involvement varied by tumour type, with higher rates in lung cancer (12.0%) and lower rates in breast cancer (4.3%) [43]. An analysis of Medicare-insured individuals with advanced cancer further demonstrated an early palliative care integration of 10.4% in 2019 [44].

Across the entire analysed cohort, among hospital cases involving patients with metastatic malignancies, and within individual medical specialties, we observed a significant positive association between inpatient mortality and the rate of specialised palliative care involvement. Evidence on the relationship between inpatient palliative care utilisation and hospital mortality remains inconsistent. On the one hand, studies have shown that patients with a high predicted risk of short-term mortality are more likely to receive palliative care consultation during hospitalisation [45]. Several other studies have identified palliative care involvement as being associated with higher in-hospital mortality and a lower likelihood of intensive care unit admission [42,46,47]. On the other hand, other studies have reported a reduction in hospital mortality associated with palliative care involvement, including a systematic review demonstrating a 34% reduction in in-hospital mortality [48,49]. A retrospective Canadian study similarly reported higher overall hospital mortality among patients receiving palliative care; however, within the subgroup of terminally ill patients receiving palliative care, the probability of dying in hospital or being admitted to an intensive care unit was lower [42].

Everett M. Rogers’ diffusion of innovations theory models how social networks absorb, evaluate, and ultimately embrace or discard new practices, distinguishing adopter groups by their timing of adoption from innovators through to laggards. Innovators and early adopters together form only about 16% of users. The 15 to 20% band beyond which diffusion typically extends into the early majority is a heuristic rather than a threshold validated for health service innovations, and in medicine it may shift further under regulatory requirements, evidence thresholds, and patient-safety considerations [11,12,13]. We therefore use it here only as a theory-derived landmark against which to read our attained utilisation rates.

Although not originally developed for healthcare, the theory is increasingly used to explain how clinicians and institutions adopt new practices, technologies, and guidelines [11,12,13]. In this setting the five innovation factors, relative advantage, compatibility, complexity, trialability, and observability, remain central but require contextual reinterpretation [12,14,15,50]. Stakeholders typically assess relative advantage in terms of clinical effectiveness, patient safety, and evidence-based outcomes. This is supported by evidence from randomised clinical trials on early integration [3,51,52,53]. Compatibility reflects alignment with professional norms, ethical standards, clinical workflows, and regulatory requirements, while complexity often relates to training demands, workflow disruption, and system interoperability [15].

Applying this model to case-based metrics requires accounting for a shift in the level of analysis. Rogers classifies individuals by their time of adoption, whereas this study analyses hospital cases [11]. A case that received palliative care does not mean the treating clinician has adopted the practice. Referrals often come from a small number of high-volume practitioners or from dedicated palliative care units, so the share of clinicians who have adopted the practice is probably lower than the share of cases receiving it [44,54,55,56]. The case-based figures reported here therefore, if anything, overstate how far the concept has spread across the treating clinician population.

Interpreted through this lens, quality-of-life-centred palliative care for patients with metastatic cancer in Germany appears largely confined to innovators and early adopters, with the early majority not yet having embraced the concept in this and previous studies. Specialised palliative care was recorded in 5.4% of all analysed cancer cases. After more than 15 years of discussion of early integration, none of our estimates exceeds the heuristic 15–20% band. Even in the metastatic subgroup, where palliative care needs are greatest, utilisation reaches only a median of 15.0% and rises to at most about 18.7% under the maximum estimate that accounts for palliative care units billing outside the DRG system. Two considerations suggest the true rate of early integration lies below even these figures. First, case-based rates tend to overstate clinician-level adoption. Second, specialised palliative care concentrated on hospital cases involving dying patients, who received this care in 31.9% of instances, indicating that late inpatient end-of-life care rather than early outpatient integration accounts for a large share of the observed services. The reported rates therefore represent an upper bound on true early integration. Even under the most favourable assumptions, utilisation does not surpass the range in which diffusion would be expected to extend into the early majority, indicating that the early majority has not yet adopted the concept.

Without broader acceptance by the early majority, specialised inpatient palliative care utilisation risks remaining a niche practice instead of becoming a normative component of oncological care. This stagnation has important implications, as it limits population-level access to timely palliative care despite strong evidence and guideline recommendations. From a diffusion and implementation science perspective, failure to convince the early majority necessitates a shift in strategy. Innovations that stall at the early-adopter stage rarely diffuse further through social influence alone and instead require structural reinforcement through policy, organisational change, and incentive realignment [11,15]. For early palliative care, this implies that continued reliance on professional advocacy and guidelines is insufficient. Instead, health systems must actively lower adoption barriers by embedding specialised inpatient palliative care utilisation and early palliative care into standard care pathways, adjusting reimbursement mechanisms, disseminating information on efficacy, raising awareness and training for health care professionals. Without such measures, specialised inpatient palliative care utilisation is unlikely to cross into early-majority adoption and may continue to be perceived as optional rather than essential.

## 5. Limitations

This study has several limitations:

1. Hospital cases do not correspond directly to individual patients, as some may be hospitalised multiple times but receive specialised inpatient palliative care only once, so case-level rates need not equal patient-level coverage. Under the German DRG rules on case merging (Fallzusammenführung), some closely spaced readmissions to the same hospital are already combined into a single case. This partly reduces multiple counting in the data. However, readmissions in a different base DRG, at a different hospital, or across the calendar-year boundary are not merged. Because the analysis is case-based, patients with advanced disease who are admitted repeatedly still contribute several cases. The resulting bias runs in two directions. First, palliative care may be coded in only some admissions, so the per-case rate can understate the share of patients ever exposed to it. Second, the same seriously ill patients are counted several times, which increases the denominator, especially in the metastatic and end-of-life subgroups. Because the second effect predominates in these subgroups, the reported rates should be read as case-level rather than patient-level figures and represent an upper bound on true patient-level coverage and early integration [57].
2. The magnitude of multiple counting cannot be derived directly, since the InEK data contain no patient identifiers. As external benchmarks, patients with advanced cancer average about 2.7 inpatient stays in the year of death, and 16% are admitted three or more times within the first year after diagnosis [57,58]. Even allowing for the partial merging described above, our 1,292,726 cases therefore represent substantially fewer unique patients, with multiple counting concentrated in the metastatic and end-of-life subgroups underlying the 15.0% figure. The reported rates therefore likely represent an upper bound.
3. The evaluation reported on hospital cases, not on health care professionals, and thus provided only indirect evidence on the percentages of innovators and early adopters. The relationship between case-level exposure and clinician-level adoption is directionally uncertain. However, if referral is concentrated among a few high-volume adopters or a dedicated consultation service, the case-exposure rate is more likely to overstate than to understate clinician-level adoption.
4. Around 70 palliative care units in Germany operate as “special facilities” (Besondere Einrichtungen), managing roughly 17,500 cases [28], and these cases would not be captured in the reimbursement database. However, some units submit data to InEK for reporting but do not bill statutory health insurance, so their activity may have been captured partially. Assuming these cases involve patients already in the analysed cohort, a sensitivity analysis yields a ceiling estimate of 6.8% overall utilisation (versus 5.4% reported) and an illustrative 18.7% for the metastatic subgroup (versus 15.0%), under the simplifying assumption of uniform distribution across entities. Even under this scenario, overall utilisation remains below the heuristic 15–20% range, and the metastatic subgroup estimate stays within it.
5. Secondary diagnoses (e.g., organ metastases coded as C79.x) may be recorded multiple times within a case, slightly affecting counts.
6. The analysis of medical specialties was restricted to disciplines commonly serving as primary treating departments in oncological centres. These listed specialties accounted for 30,701 of the 70,382 total specialised palliative care cases (43.6%); the remaining cases involved departments not included in this categorisation. Because specialty assignment is non-disjoint, a case counted within one specialty’s exposed group may also appear within another specialty’s remainder group. Odds ratios for medical specialties should therefore be interpreted as reflecting the relative frequency of specialised palliative care involvement per specialty contact, rather than as fully independent comparisons.
7. The InEK dataset does not capture the timing of palliative care referral relative to diagnosis. Consequently, we cannot distinguish early palliative care integration from palliative care initiated shortly before death.
8. Because specialty selection captures all departments with which a case had contact rather than a single assigned department, cases involving cross-specialty co-management or internal transfer are counted within each contributing specialty. This non-disjoint assignment means that specialty stratified totals cannot be summed to reconstruct the overall case count and should be interpreted as reflecting specialty involvement rather than exclusive attribution.
9. Suppression of codes representing fewer than four cases removed the smallest subgroups from the dataset, with unquantifiable effects on rare tumour entities or procedures.
10. Because odds ratios were calculated from aggregated tables rather than individual patient records, they represent crude, unadjusted associations. Individual-level logistic regression was not feasible given the aggregated nature of the InEK output.
11. The linear regressions represent ecological correlations, and inference at the patient level would risk an ecological fallacy. As the regressions were unweighted, entities with small case numbers exerted the same influence as those with larger case numbers, which may affect the stability of the estimates.
12. Hospital mortality was calculated per case without specifying cause but remains a relevant indicator of disease severity.
13. Outpatient care is not included. Patient may receive specialised outpatient palliative care after discharge. These data are not available.
14. Findings rely on accurate coding in the InEK database. Misclassification may introduce bias.
15. Finally, the dataset lacks information on symptom burden, psychosocial stress, caregiver experience, and quality of life. The study therefore measures coded inpatient utilisation only, with specialised palliative care coding serving as an administrative proxy for utilisation rather than a measure of clinical benefit. It cannot assess the quality, timing, or effectiveness of the care actually delivered.

## 6. Conclusions

Despite longstanding guideline recommendations, specialised inpatient palliative care remains underutilised in German oncology, reaching 5.4% of all analysed cases and a median of 15.0% even in the metastatic subgroup. Interpreted cautiously through the diffusion of innovations theory, which treats the 15 to 20% band as a heuristic landmark rather than a validated threshold, utilisation appears confined to innovators and early adopters, with the early majority not yet reached. Because these are case-based rather than clinician-level rates, and because much of the coded activity clusters around end-of-life admissions, the figures likely represent an upper bound on true early integration. These findings identify a persistent adoption gap and suggest that structural approaches, including systematic referral pathways, aligned incentives, and expanded capacity, merit prospective evaluation as potential ways to move uptake beyond early adopters.

## Figures and Tables

**Table 1:** Age and gender distribution.

**All German Hospital Cases**																										
**Tumour Entities**	**Oral Cavity/Throat**		**Stomach**		**Intenstine/Rectum**		**Pancreas**		**Lung**		**Malignant melanoma**		**Breast**		**Cervix**		**Uterus (without cervix)**		**Ovary**		**Prostate**		**Kidney**		**Bladder**	
defining CD-10-GM code	C00–14		C16		C18–20		C25		C34		C43		C50		C53		C54–C55		C56		C61		C64		C67	
	hospital cases	%	hospital cases	%	hospital cases	%	hospital cases	%	hospital cases	%	hospital cases	%	hospital cases	%	hospital cases	%	hospital cases	%	hospital cases	%	hospital cases	%	hospital cases	%	hospital cases	%
**all hospital cases**	64,973		55,714		181,464		77,490		254,444		44,705		207,681		18,215		27,961		35,523		158,001		37,420		129,135	
**gender**																										
male	46,347	71.3%	36,187	65.0%	105,608	58.2%	40,298	52.0%	146,114	57.4%	24,759	55.4%	2087	1.0%	1	0.0%	4	0.0%	8	0.0%	157,964	100.0%	25,146	67.2%	99,951	77.4%
female	18,623	28.7%	19,521	35.0%	75,850	41.8%	37,181	48.0%	108,312	42.6%	19,944	44.6%	205,580	99.0%	18,214	100.0%	27,957	100.0%	35,513	100.0%	32	0.0%	12,273	32.8%	29,178	22.6%
diverse	1	0.0%	0	0.0%	1	0.0%	0	0.0%	0	0.0%	1	0.0%	1	0.0%	0	0.0%	0	0.0%	0	0.0%	0	0.0%	0	0.0%	3	0.0%
unknown	2	0.0%	6	0.0%	5	0.0%	11	0.0%	18	0.0%	1	0.0%	13	0.0%	0	0.0%	0	0.0%	2	0.0%	5	0.0%	1	0.0%	3	0.0%
**age groups**																										
16–17 years	295	0.5%	209	0.4%	783	0.4%	109	0.1%	155	0.1%	511	1.1%	826	0.4%	188	1.0%	49	0.2%	669	1.9%	14	0.0%	129	0.3%	105	0.1%
18–29 years	726	1.1%	1010	1.8%	2738	1.5%	423	0.5%	1066	0.4%	1720	3.8%	8313	4.0%	2300	12.6%	392	1.4%	1118	3.1%	27	0.0%	436	1.2%	588	0.5%
30–39 years	2997	4.6%	2919	5.2%	8151	4.5%	2251	2.9%	5487	2.2%	3010	6.7%	22,868	11.0%	3562	19.6%	1287	4.6%	2424	6.8%	622	0.4%	1485	4.0%	2295	1.8%
40–49 years	4594	7.1%	3297	5.9%	10,244	5.6%	3544	4.6%	10,628	4.2%	2782	6.2%	19,853	9.6%	2075	11.4%	1750	6.3%	2778	7.8%	2700	1.7%	1864	5.0%	3692	2.9%
50–54 years	9057	13.9%	5441	9.8%	16,968	9.4%	7421	9.6%	25,669	10.1%	4454	10.0%	23,524	11.3%	2407	13.2%	3259	11.7%	4162	11.7%	8736	5.5%	3519	9.4%	8519	6.6%
55–59 years	12,569	19.3%	7466	13.4%	22,942	12.6%	10,449	13.5%	43,090	16.9%	4984	11.1%	25,799	12.4%	2377	13.0%	4318	15.4%	5093	14.3%	17,527	11.1%	5139	13.7%	14,246	11.0%
60–64 years	21,335	32.8%	16,788	30.1%	53,800	29.6%	25,991	33.5%	101,012	39.7%	10,641	23.8%	49,260	23.7%	3349	18.4%	8579	30.7%	9825	27.7%	58,657	37.1%	12,449	33.3%	40,405	31.3%
65–74 years	6002	9.2%	6979	12.5%	23,463	12.9%	11,380	14.7%	33,273	13.1%	5467	12.2%	20,503	9.9%	797	4.4%	3161	11.3%	3833	10.8%	26,241	16.6%	4952	13.2%	19,787	15.3%
75–79 years	7398	11.4%	11,605	20.8%	42,375	23.4%	15,922	20.5%	34,064	13.4%	11,136	24.9%	36,735	17.7%	1160	6.4%	5166	18.5%	5621	15.8%	43,477	27.5%	7447	19.9%	39,498	30.6%
**fatal cases**																										
**tumour entities**	**oral cavity/throat**		**stomach**		**intenstine/rectum**		**pancreas**		**lung**		**malignant melanoma**		**breast**		**cervix**		**uterus (without cervix)**		**ovary**		**prostate**		**kidney**		**bladder**	
defining CD-10-GM code	C00–14		C16		C18–20		C25		C34		C43		C50		C53		C54–C55		C56		C61		C64		C67	
	hospital cases	%	hospital cases	%	hospital cases	%	hospital cases	%	hospital cases	%	hospital cases	%	hospital cases	%	hospital cases	%	hospital cases	%	hospital cases	%	hospital cases	%	hospital cases	%	hospital cases	%
**all hospital cases**	3045		4304		10,595		8264		23,715		1591		8134		727		1261		2243		6894		2139		4113	
		4.7%		7.7%		5.8%		10.7%		9.3%		3.6%		3.9%		4.0%		4.5%		6.3%		4.4%		5.7%		3.2%
**gender**																										
male	2273	74.6%	2834	65.8%	6259	59.1%	4451	53.9%	14,464	61.0%	1015	63.8%	103	1.3%	0	0.0%	0	0.0%	1	0.0%	6888	99.9%	1476	69.0%	2987	72.6%
female	771	25.3%	1468	34.1%	4333	40.9%	3807	46.1%	9245	39.0%	575	36.1%	8028	98.7%	727	100.0%	1261	100.0%	2240	99.9%	2	0.0%	662	30.9%	1125	27.4%
diverse	0	0.0%	0	0.0%	0	0.0%	0	0.0%	0	0.0%	0	0.0%	0	0.0%	0	0.0%	0	0.0%	0	0.0%	0	0.0%	0	0.0%	0	0.0%
unknown	1	0.0%	2	0.0%	3	0.0%	6	0.1%	6	0.0%	1	0.1%	3	0.0%	0	0.0%	0	0.0%	2	0.1%	4	0.1%	1	0.0%	1	0.0%
**age groups**																										
16–17 years	4	0.1%	15	0.3%	16	0.2%	3	0.0%	8	0.0%	5	0.3%	11	0.1%	2	0.3%	0	0.0%	15	0.7%	0	0.0%	1	0.0%	0	0.0%
18–29 years	10	0.3%	60	1.4%	79	0.7%	20	0.2%	64	0.3%	37	2.3%	171	2.1%	49	6.7%	11	0.9%	21	0.9%	0	0.0%	11	0.5%	16	0.4%
30–39 years	67	2.2%	158	3.7%	258	2.4%	141	1.7%	349	1.5%	68	4.3%	537	6.6%	116	16.0%	39	3.1%	80	3.6%	11	0.2%	40	1.9%	39	0.9%
40–49 years	155	5.1%	190	4.4%	358	3.4%	270	3.3%	707	3.0%	81	5.1%	472	5.8%	75	10.3%	41	3.3%	110	4.9%	38	0.6%	69	3.2%	82	2.0%
50–54 years	311	10.2%	335	7.8%	650	6.1%	630	7.6%	1822	7.7%	140	8.8%	706	8.7%	84	11.6%	100	7.9%	203	9.1%	129	1.9%	143	6.7%	156	3.8%
55–59 years	501	16.5%	501	11.6%	952	9.0%	887	10.7%	3386	14.3%	171	10.7%	854	10.5%	92	12.7%	146	11.6%	258	11.5%	328	4.8%	219	10.2%	307	7.5%
60–64 years	1077	35.4%	1221	28.4%	2795	26.4%	2548	30.8%	9164	38.6%	354	22.3%	1969	24.2%	151	20.8%	401	31.8%	645	28.8%	1619	23.5%	633	29.6%	1059	25.7%
65–74 years	371	12.2%	555	12.9%	1509	14.2%	1286	15.6%	3542	14.9%	221	13.9%	956	11.8%	56	7.7%	175	13.9%	279	12.4%	1194	17.3%	297	13.9%	641	15.6%
75–79 years	549	18.0%	1269	29.5%	3978	37.5%	2479	30.0%	4673	19.7%	514	32.3%	2458	30.2%	102	14.0%	348	27.6%	632	28.2%	3575	51.9%	726	33.9%	1813	44.1%

**Table 2:** Rate of specialised palliative care treatment in the analysed tumours for all hospital and fatal cases.

**All Hospital Cases**																														
**Location of Solid Tumours**		**Location of Solid Tumours**	**Oral Cavity & Throat**		**Stomach**		**Intestine/rectum**		**Pancreas**		**Lung**		**Malignant melanoma**		**Breast**		**Cervix**		**Uterus without cervix**		**Ovary**		**Prostate**		**Kidney**		**Urinary bladder**		**Total**	
ICD-10 code		ICD-10 code	C00–14		C16		C18–20		C25		C34		C43		C50		C53		C54–55		C56		C61		C64		C67			
**total number of patients**			**64,973**		**55,714**		**181,464**		**77,490**		**254,444**		**44,705**		**207,681**		**18,215**		**27,961**		**35,523**		**158,001**		**37,420**		**129,135**		1,292,726	
received no PC			62,187	95.7%	51,914	93.2%	172,640	95.1%	69,659	89.9%	234,599	92.2%	43,077	96.4%	198,374	95.5%	17,097	93.9%	26,560	95.0%	32,291	90.9%	152,343	96.4%	35,351	94.5%	126,252	97.8%	1,222,344	94.6%
number cases receiving SPC			2786	4.3%	3800	6.8%	8824	4.9%	7831	10.1%	19,845	7.8%	1628	3.6%	9307	4.5%	1118	6.1%	1401	5.0%	3232	9.1%	5658	3.6%	2069	5.5%	2883	2.2%	70,382	5.4%
palliative care codes	total	OPS code	2896		3920		9060		8047		20,509		1708		9582		1170		508		3348		5806		2145		2999		71,698	
	complex palliative care (e.g., on an external ward)	8-982	698	25.1%	1230	32.4%	2965	33.6%	2493	31.8%	6567	33.1%	421	25.9%	2959	31.8%	289	25.8%	124	8.9%	938	29.0%	1768	31.2%	665	32.1%	800	27.7%	21,917	31.1%
	specialised palliative care on a palliative care ward	8-98e	1269	45.5%	1841	48.4%	4381	49.6%	3981	50.8%	9602	48.4%	751	46.1%	4571	49.1%	516	46.2%	292	20.8%	1544	47.8%	2942	52.0%	1047	50.6%	1482	51.4%	34,219	48.6%
	specialised palliative care by a consultation service	8-98h	929	32.1%	849	21.7%	1714	18.9%	1573	19.5%	4340	21.2%	536	31.4%	2052	21.4%	365	31.2%	92	18.1%	866	25.9%	1096	18.9%	433	20.2%	717	23.9%	15,562	21.7%
**fatal hospital cases**																														
all hospital cases																														
**location of solid tumours**		**location of solid tumours**	**oral cavity & throat**		**stomach**		**intestine/rectum**		**pancreas**		**lung**		**malignant melanoma**		**breast**		**cervix**		**uterus without cervix**		**ovary**		**prostate**		**kidney**		**urinary bladder**		**total**	
ICD-10 code		ICD-10 code	C00–14		C16		C18–20		C25		C34		C43		C50		C53		C54–55		C56		C61		C64		C67			
**total number of patients**			**3045**		**4304**		**10,595**		**8264**		**23,715**		**1591**		**8134**		**727**		**1261**		**2243**		**6894**		**2139**		**4113**		77,025	
received no PC			2145	70.4%	2649	61.5%	7627	72.0%	5258	63.6%	16,650	70.2%	978	61.5%	5217	64.1%	432	59.4%	789	62.6%	1290	57.5%	5039	73.1%	1443	67.5%	2942	71.5%	52,459	68.1%
number cases receiving SPC			900	29.6%	1655	38.5%	2968	28.0%	3006	36.4%	7065	29.8%	613	38.5%	2917	35.9%	295	40.6%	472	37.4%	953	42.5%	1855	26.9%	696	32.5%	1171	28.5%	24,566	31.9%
palliative care codes	total	OPS code	1102				3093		3119		8079		649		3080		290		508		1008		1933		577		1235		24,673	
	complex palliative care (e.g., on an external ward)	8-982	536	59.6%	395	23.9%	856	28.8%	833	27.7%	2101	29.7%	156	25.4%	777	26.6%	69	23.4%	124	26.3%	241	25.3%	451	24.3%	80	11.5%	267	22.8%	6886	28.0%
	specialised palliative care on a palliative care ward	8-98e	333	37.0%	883	53.4%	1856	62.5%	1853	61.6%	4782	67.7%	371	60.5%	1830	62.7%	189	64.1%	292	61.9%	603	63.3%	1221	65.8%	447	64.2%	757	64.6%	15,417	62.8%
	specialised palliative care by a consultation service	8-98h	233	21.1%	210	#DIV/0!	381	12.3%	433	13.9%	1196	14.8%	122	18.8%	473	15.4%	32	11.0%	92	18.1%	164	16.3%	261	13.5%	50	8.7%	211	17.1%	3858	15.6%

## Data Availability

The data underlying this study were obtained from the InEK Datenbrowser (https://datenbrowser.inek.org/), accessed on 12 November 2025. The extracted case-level tables and analysis files are available from the corresponding author upon reasonable request.

## Supplementary Materials

The following supporting information can be downloaded at: https://www.mdpi.com/article/10.3390/cancers18193163/s1, Figure S1: Different types of specialised palliative care across different tumour types; Figure S2: Odds ratios for specialised palliative care utilisation for different comorbidities. This figure presents odds ratios for specialised palliative care treatment according to comorbidities. Odds ratios are calculated relative to the overall frequency of specialised palliative care utilisation in the entire analysed cohort. Abbreviations: *except hemiplegia. Table S1: Code definitions and query parameters. Code definitions and query parameters used for case identification. OPS codes for specialised palliative care, ICD-10-GM codes defining the analysed tumour entities and metastatic sites (as previously listed), selected age groups, medical specialties with corresponding department keys (Fachabteilungsschlüssel), and discharge reason codes (§301 SGB V) used to identify all hospital cases and fatal hospital cases (discharge reason 07: death); Table S2: Comorbidities according to the Charlson Comorbidity Index; Table S3: In-hospital mortality in the entire cohort and in hospital cases involving patients with metastases; Table S4: Calculated odds ratios for inpatient palliative care.
